# Brazilian Academy of Neurology recommendations for diagnosis, management, and treatment of chronic inflammatory demyelinating polyradiculoneuropathy (CIDP)

**DOI:** 10.1055/s-0045-1809884

**Published:** 2025-07-28

**Authors:** Osvaldo J. M. Nascimento, Wilson Marques Jr, Marcus Vinícius Magno Gonçalves, Pedro José Tomaselli, Camila Pupe, Marcondes Cavalcante França Jr, Francisco de Assis Aquino Gondim, Marcos Raimundo Gomes de Freitas, Rodrigo Siqueira Soares Frezatti, Acary Souza Bulle Oliveira, Francisco Tellechea Rotta, Elza Dias Tosta, Rosana Scola, Vanessa Daccach, Carlo Domenico Marrone, Jefferson Becker, Susanie Rigatto, Alberto R. M. Martinez, Hideraldo Cabeça, Pablo Brea Winckler, Mario Emilio Dourado, Diogo Fernandes dos Santos

**Affiliations:** 1 Universidade Federal Fluminense, Faculdade de Medicina, Niterói RJ, Brazil.; 2 Universidade de São Paulo, Faculdade de Medicina de Ribeirão Preto, Ribeirão Preto SP, Brazil.; 3 Universidade de Joinville, Joinville SC, Brazil.; 4 Universidade de Campinas, Faculdade de Ciências Médicas, Campinas SP, Brazil.; 5 Universidade Federal do Ceará, Faculdade de Medicina, Fortaleza CE, Brazil.; 6 Universidade Federal de São Paulo, Escola Paulista de Medicina, São Paulo SP, Brazil.; 7 Hospital Moinhos de Vento, Porto Alegre RS, Brazil.; 8 Santa Casa de Misericórdia de Porto Alegre, Porto Alegre RS, Brazil.; 9 Hospital dos Servidores da União de Brasília, Brasília DF, Brazil.; 10 Universidade Federal do Paraná, Curitiba PR, Brazil.; 11 Clinica Marrone, Porto Alegre, RS, Brazil.; 12 Pontifícia Universidade Católica do Rio Grande do Sul, Escola de Medicina, Instituto do Cérebro, Porto Alegre RS, Brazil.; 13 Hospital Geral de Goiânia, Goiânia GO, Brazil.; 14 Hospital Geral de Belém, Belém PA, Brazil.; 15 Universidade Federal do Rio Grande do Sul, Faculdade de Medicina, Porto Alegre RS, Brazil.; 16 Universidade Federal do Rio Grande do Norte, Faculdade de Medicina, Natal RN, Brazil.; 17 Universidade Federal de Uberlândia, Faculdade de Medicina, Uberlândia MG, Brazil.

**Keywords:** Polyradiculoneuropathy, Chronic Inflammatory Demyelinating, Consensus, Diagnosis, Therapeutics

## Abstract

Chronic inflammatory demyelinating polyradiculoneuropathy (CIDP) is an acquired autoimmune disorder that leads to progressive motor and sensory impairment, resulting in significant morbidity. While the incidence rates vary, CIDP remains a challenging condition requiring a standardized and optimized approach to diagnosis and management. In Brazil, a middle-income country with substantial regional disparities in healthcare access, the availability of specialized neuromuscular centers is uneven, creating obstacles to timely and effective treatment. To address these challenges, the Brazilian Academy of Neurology (Academia Brasileira de Neurologia, ABN, in Portuguese) has developed national recommendations for the diagnosis, management, and treatment of CIDP, tailored to the country's healthcare resources. This consensus outlines standardized diagnostic criteria that incorporate electrophysiological and imaging findings, and it emphasizes key differential diagnoses to enhance diagnostic accuracy. The recommendations were developed through expert panel discussions and a non-systematic review of the literature. The recommended treatment strategies include first-line therapies such as corticosteroids, intravenous immunoglobulin (IVIg), and plasmapheresis, with guidance on escalation and titration of immunosuppressive therapy in refractory cases. By emphasizing early intervention to prevent axonal degeneration and disability, these guidelines aim to improve clinical outcomes and support public health policies within the Brazilian National Health System (Sistema Único de Saúde, SUS, in Portuguese), ensuring equitable and effective CIDP management across the country.

## INTRODUCTION

Chronic inflammatory demyelinating polyradiculoneuropathy (CIDP) is a rare acquired, autoimmune, demyelinating peripheral neuropathy. It classically presents with a progressive course throughout more than 8 weeks and is characterized by symmetrical motor predominant impairment affecting both distal and proximal regions of the upper and lower limbs and associated with areflexia. However, up to 16% of the patients may experience a more rapid progression, with symptoms developing over a shorter period (< 4 weeks), a presentation known as acute-onset CIDP, which can resemble Guillain-Barré syndrome (GBS). Mild autonomic dysfunction is common but is typically less severe than in GBS, and respiratory and cranial nerve involvement are not frequent. 1

Electrophysiological findings support peripheral nerve demyelination, evidenced by conduction block (CB), temporal dispersion (TD), abnormal F wave, and non-uniform slowing of conduction velocity (CV), along with albumin-cytologic dissociation. 2

Nearly 50 years have passed since the initial report by Dyck et al. 3 detailing the clinical presentation, the electrophysiological findings, the characteristics of nerve biopsy, and the response to the corticosteroid treatment. During this period, several criteria for the diagnosis of CIDP have been proposed, 4 5 6 with most emphasizing clinical presentations and nerve conduction studies (NCSs) as keys components of the diagnosis. 7 8 However, the correct diagnosis remains challenging, and a significant number of patients worldwide are misdiagnosed and improperly treated. 9 10 11

The incidence and prevalence of CIDP vary significantly among different populations, influenced by differences in diagnostic criteria and study methodologies. Incidence rates have been reported between 0.33 and 0.68 per 100 thousand person-years, while prevalence rates range from 1.61 to 8.9 per 100 thousand individuals. 12 13 14 15 These variations reflect the impact of geographic, demographic, and methodological factors from different studies.

The condition has been a topic of interest among Brazilian neurologists for 30 years. In 1995, a study 16 involving 45 cases, predominantly of the typical CIDP, was conducted in Rio de Janeiro. Subsequently, in 1997, an analysis 17 of 18 cases was carried out in São Paulo. In 2013, a comparative epidemiological study 18 involving North-American and Brazilian peripheral nerve centers analyzed 1,090 and 1,034 cases, respectively. Among these, immune-mediated neuropathies accounted for 215 (19.7%) and 191 (18%) cases, with CIDP representing 107 (∼ 50%) and 116 (∼ 61%) of these cases, respectively. Chronic inflammatory demyelinating polyradiculoneuropathy significantly impairs the quality of life (QoL) of Brazilian patients. 19

In Brazil, a middle-income country of continental dimensions, neuromuscular centers with neurologists with expertise in the diagnosis and treatment of CIDP are unevenly distributed across the country. This disparity highlights the urgent need for a standardized approach to ensure equitable access, optimize diagnosis, and improve treatment outcomes nationwide.

The aim of the present study was to establish recommendations, under the guidance of the Scientific Department of Peripheral Neuropathy of the Brazilian Academy of Neurology (Academia Brasileira de Neurologia, ABN, in Portuguese), to standardize the diagnosis, management, and treatment of CIDP considering local resources.

Such a consensus is crucial not only to improve clinical outcomes but also to inform public health policies within the Brazilian National Health System (Sistema Único de Saúde, SUS, in Portuguese). This initiative is expected to serve as a cornerstone to improve care delivery and ensure the equitable management of CIDP throughout Brazil.

## METHODS

A group of 22 Brazilian neurologists with expertise in peripheral neuropathy, all active members of the ABN's Scientific Department of Peripheral Neuropathy, collaborated to develop the current consensus. Each member contributed by summarizing their individual perspectives on key aspects of the topic, which were then collectively reviewed and refined through an iterative process. Disagreements among members were resolved through iterative discussions, ensuring a balanced and well-supported consensus.

A non-systematic review of the current literature on CIDP was conducted to synthesize evidence on its diagnosis and treatment. A comprehensive search was performed using the PubMed, SciELO, and Embase databases to identify relevant studies. The inclusion criteria encompassed peer-reviewed journal articles focusing on CIDP diagnosis and treatment, while case reports, non-peer-reviewed sources, and articles not published in English or Portuguese were excluded. No formal grading system was applied for evidence evaluation; however, consensus was established based on the best available clinical guidelines and expert recommendations to ensure a rigorous and informed synthesis of the literature.

The manuscript was structured as a questionnaire, with questions designed to address critical aspects of CIDP diagnosis, differential diagnosis, common pitfalls, and treatment challenges. The aim was to clarify real-world clinical challenges and establish evidence-based practices within the context of the resources available. The final guidelines were reviewed to align with current evidence-based practices.

## RESULTS

### Difficulty in establishing a correct diagnosis?

Several factors contribute to high rates of CIDP misdiagnosis, making it a significant problem in the clinical practice worldwide, as patients may receive inappropriate treatments that may delay proper management and worsen outcomes. Studies from Serbia 11 and the United States and the Netherlands 10 found that 52%, 32%, and 47% of the cases referred with a diagnosis of CIDP, respectively, were overdiagnosed. These findings highlight the widespread difficulty in correctly diagnosing CIDP, particularly its variants, which can present overlapping features with other neuropathies, leading to incorrect diagnoses. 20

One contributing factor is the clinicians' overreliance on electromyography (EMG) reports without considering the full clinical picture. This can lead to a high rate of false positives, as NCSs may present in CIPD the same pattern seen in others disorders, such as leprosy, hereditary transthyretin amyloidosis (hATTR) and others inherited neuropathies. 21 Interestingly, concomitance of inherited neuropathies and CIDP has been reported. 23

Another common contributor to misdiagnosis is reeling diagnosis on albuminocytological dissociation in the cerebrospinal fluid (CSF) as a primary diagnostic criterion. Up to 50% of patients without CIDP can present with mild protein elevation 24. In such cases, protein elevation is typically mild. 24 In a study by Allen and Bril, 25 albuminocytological dissociation was present in 50% of the misdiagnosed patients, but only in 2 the level was greater than 100 mg/dL. Lumbar puncture is not necessary if patients fulfill the clinical and electrophysiological criteria. However, it should be performed in patients with ‘atypical’ features. 26 Pleocytosis of the CSF should raise suspicion for alternative diagnoses such as lymphomatous infiltration, other malignancies, neurosarcoidosis, or infectious etiologies.

Additionally, it is not uncommon for patients to report improvement after receiving immunotherapy, even when the diagnosis of CIDP was incorrect. Thus, objective indicators of treatment efficacy are essential in order to make informed treatment decisions.

The European Academy of Neurology/Peripheral Nerve Society (EAN/PNS) Grading of Recommendations Assessment, Development and Evaluation (GRADE) Guidelines on diagnosis and treatment of CIDP considered that electrodiagnosis is strongly recommended, 2 although introducing changes to the previous criteria, 6 considering only two possibilities: CIDP and possible CIDP ( Tables 1 , 2 ).

**Table 1:** Clinical criteria for CIDP

**Typical CIDP**
**A+ B + C**
**A:** Progressive or relapsing, symmetric, proximal, and distal muscle weakness of the upper and lower limbs, and sensory involvement of at least two limbs. **B:** Developing over at least 8 weeks. **C:** Absent or reduced tendon reflexes in all limbs.
**CIDP variants**
One of the following:
**Distal CIDP:** distal sensory loss and muscle weakness predominantly in the lower limbs.
**Multifocal CIDP:** sensory loss and muscle weakness in a multifocal pattern (at least two nerves), usually asymmetric, and upper limb predominant.
**Focal CIDP:** sensory loss and muscle weakness only in one nerve.
**Motor CIDP:** motor symptoms and signs without sensory involvement.
**Sensory CIDP:** sensory symptoms and signs without motor involvement.

Abbreviation: CIDP, chronic inflammatory demyelinating polyradiculoneuropathy.

Note: Adapted from Van den Bergh et al., 2021. ^2^

**Table 2:** Diagnostic categories for CIDP

**Typical CIDP**	• Clinical criteria + motor conduction criteria in two nerves + sensory conduction abnormalities in two nerves.
	• Possible typical CIDP + at least two supportive criteria.
**Possible typical CIDP**	• Clinical criteria + motor conduction criteria in one nerve + sensory conduction abnormalities in two nerves.
	• Clinical criteria + motor conduction abnormalities not fulfilling CIDP motor conduction criteria in one nerve + sensory conduction abnormalities in two nerves + objective response to treatment + one other supportive criterion.
**Distal CIDP**	• Clinical criteria + motor conduction criteria in two upper limb nerves + sensory conduction abnormalities in two nerves.
	• Possible distal CIDP + at least two supportive criteria.
**Possible distal CIDP**	• Clinical criteria + motor conduction criteria in one upper limb nerve + sensory conduction abnormalities in one nerve.
	• Clinical criteria + motor conduction criteria in two lower limb nerves only + sensory conduction abnormalities in two nerves (possible distal CIDP only, cannot be upgraded by supportive criteria).
**Multifocal or focal CIDP**	• Clinical criteria + motor conduction criteria in two nerves + sensory conduction abnormalities in two nerves.
	• Possible multifocal or focal CIDP + at least two supportive criteria.
**Possible multifocal or focal CIDP**	• Clinical criteria + motor conduction criteria in one nerve + sensory conduction abnormalities in two nerves.
	• Focal CIDP fulfilling the clinical criteria + motor conduction criteria in one nerve + sensory conduction abnormalities in one nerve (possible focal CIDP only, cannot be upgraded by supportive criteria).
**Motor CIDP**	• Clinical criteria + motor conduction criteria in two nerves + normal sensory conduction in four nerves.
	• Possible motor CIDP + at least two supportive criteria.
**Possible motor CIDP**	• Clinical criteria + motor conduction criteria in one nerve + normal sensory conduction in four nerves.
**Motor-predominant CIDP**	• As in motor CIDP, but with sensory conduction abnormalities in two nerves.
**Sensory CIDP**	
**Possible sensory CIDP**	• Clinical criteria + sensory conduction criteria (possible sensory CIDP only, cannot be upgraded by supportive criteria). Motor conduction must be normal in at least four nerves.
Sensory-predominant CIDP	
**Possible sensory-predominant CIDP**	• Clinical criteria + sensory conduction abnormalities in two nerves + motor conduction abnormalities in two nerves or motor conduction criteria fulfilment in one nerve.
**Sensory-predominant CIDP**	• Clinical criteria + sensory conduction abnormalities in two nerves + motor conduction criteria fulfilment in two nerves.

Abbreviation: CIDP, chronic inflammatory demyelinating polyradiculoneuropathy.

Note: Adapted from Van den Bergh et al., 2021. ^2^

### EMG criteria

Additionally, they 2 included abnormalities in sensory conduction that had not been previously considered. The proposed electrodiagnostic criteria are presented in Table 3 , and the specific motor and sensory criteria are presented in Tables 4 and 5 , respectively. Abnormalities on EMG enormously contribute to the diagnosis of CIDP and its variants, but they are not specific and should be evaluated considering the clinical context.

**Table 3:** Electrodiagnostic criteria

Disease type	Number of abnormal nerves		Observation
	MNC	SNC	
CIDP	≥ 2	≥ 2	
Possible CIDP ^*^	1	1	Should fulfill clinical criteria, respond to treatment, and have an additional supportive criterion
Distal CIDP	≥ 2 ULiN	≥ 2	
Possible distal CIDP	1 ULiN or 2 LLiN	≥ 2	
Multifocal CIDP	≥ 2 ^*^	≥ 2 ^*^	
Possible Multifocal CIDP	1	1	≥ 2 limbs affected
Possible Focal CIDP	1	1	
Motor CIDP	≥ 2	Normal*	*SNC should be normal in at least 4 nerves (median, ulnar, radial, sural)
Possible motor CIDP	1	Normal*	*SNC should be normal in at least 4 nerves (median, ulnar, radial, sural)
Motor-predominant CIDP	≥ 2	≥ 2	Clinically-motor CIDP with abnormalities in SNC
Sensory CIDP	Normal*	≥ 2	*MNC should be normal in at least 4 nerves (median, ulnar, tibial, peroneal)
Possible sensory-predominant CIDP	1	≥ 2	Clinically-sensory CIDP with abnormalities in SNC
Sensory-predominant CIDP	≥ 2	≥ 2	Clinically-sensory CIDP with abnormalities in SNC

Abbreviations: CIDP, chronic inflammatory demyelinating polyradiculoneuropathy; LLiN, lower-limb nerves; MNC, motor nerve conduction; SNC, sensory nerve conduction; ULiN, upper-limb nerves.

Note: Adapted from Van den Bergh, 2021. ^2^

**Table 4:** Motor conduction criteria

Criteria	Observation
Motor distal latency prolongation > 50% above ULN in ≥ 2 nerves	Excluding the median nerve at the wrist in cases of carpal tunnel syndrome
Reduction of motor conduction velocity > 30% below LLN in in ≥ 2 nerves	
Prolongation of F-wave latency > 20% above ULN in ≥ 2 nerves	> 50% if amplitude of distal negative peak CMAP < 80% of LLN
Absence of F-waves in 2 nerves + ≥ 1 other demyelinating parameter in another nerve	If distal negative peak CMAP amplitudes > 20% of LLN
Motor conduction block in 2 nerves or 1 nerve + > 1 other demyelinating parameter in another nerve (excluding F-wave criteria)	> 30% reduction in the negative peak CMAP amplitude, excluding the tibial nerve, and distal negative peak CMAP amplitude > 20% of LLN
Abnormal temporal dispersion	> 30% duration increase between the proximal and distal negative peak CMAP. 100% in the tibial nerve
Distal CMAP duration (median: > 8.4 ms; ulnar: > 9.6 ms; peroneal: > 8.8 ms; and tibial: > 9.2 ms) + > 1 other demyelinating parameter in > 1 other nerve	Interval between onset of the first negative peak and return to baseline of the last negative peak

Abbreviations: CMAP, compound muscle action potential; LLN, lower limit of normal; ULN, upper limit of normal.

Notes: Strongly supportive of demyelination: at least one criterion; Weakly supportive of demyelination: abnormalities only in one nerve. Frequency filter bandpass from 2 Hz to 10kHz. Adapted from Van den Bergh, 2021. ^2^

**Table 5:** Sensory conduction criteria

Disease	Criteria
CIDP	Sensory conduction abnormalities (prolonged distal latency, or reduced SNAP amplitude, or slowed conduction velocity outside of normal limits) in 2 nerves
Possible CIDP	Abnormalities in 1 nerve
Sensory CIDP	Sensory nerve conduction velocity < 80% of LLN (for SNAP amplitude > 80% of LLN) or < 70% of LLN (for SNAP amplitude < 80% of LLN) in at least 2 nerves (median, ulnar, radial, sural nerve), or sural sparing pattern (abnormal median or radial SNAP with normal sural SNAP, excluding carpal tunnel syndrome)

Abbreviations: CIDP, chronic inflammatory demyelinating polyradiculoneuropathy; LLN, lower limit of normal; SNAP, sensory nerve action potential; ULN, upper limit of normal.

Notes: Strongly supportive of demyelination: at least one criterion; Weakly supportive of demyelination: abnormalities only in one nerve. Frequency filter bandpass from 2 Hz to 10kHz. Adapted from Van den Bergh, 2021. ^2^

### Early diagnosis and diagnostic criteria?

Early diagnosis is crucial to avoid secondary axonal damage. Previous criteria, such as those established by the American Academy of Neurology (AAN) 5 and the Rotterdam Inflammatory Neuropathy Cause and Treatment (INCAT) Group, 27 were primarily designed for research purposes. While the ANN criteria present high specificity (100%), they suffer from low sensitivity (45.7%) for the diagnosis of CIDP. 28 In 2010, the European Federation of Neurology Societies and the PNS (EFNS/PNS) guideline ^6^ introduced updated criteria, which were further refined in 2021 by the EAN/PNS; 2 these criteria increased the specificity and sensibility of CIDP diagnosis and incorporated peripheral nerve system (PNS) imaging as an additional diagnostic tool. Another significant advancement was the redefinition of antibody-mediated autoimmune nodo-paranodopathies (neurofascin-155, contactin-1, and contactin-associated protein 1 [Caspr1]), 29 which used to be considered variants of CIDP. 30 31 These conditions represent a completely distinct clinical entity, with clinical and pathophysiological features that differ significantly from those of typical CIDP and require specific therapeutic approaches. 2

This new diagnostic framework includes “CIDP” and “possible CIDP”, eliminating the classification of “probable CIDP” and “definite CIDP” 2 ( Table 2 ) used in the AAN and INCAT criteria. Additionally, clinical and electrodiagnostic criteria for typical CIDP and its variants, which include distal, multifocal/focal, motor, and sensory CIDP, were defined. Analysis of the CSF has become a supportive criterion, alongside PNS imaging, including techniques such as ultrasonography (US), magnetic resonance imaging (MRI), and neurography to evaluate the spinal roots, the brachial or lumbosacral plexuses, and/or individual nerves ( Table 6 ). This is usually reserved for atypical cases, often when the clinical and electrophysiological findings are focal (such as in multifocal CIDP) or exclusively motor and are used to support the diagnosis when enlargements of nerve roots are found or to rule out other diagnoses such as of an infiltrative pathology. 25 High-resolution US seems to be more useful than MRI in the study of the proximal structures of peripheral nerves. 2

**Table 6:** Suggested propedeutics according to clinical suspicion

When	Investigation
**Initial approach**	Electrodiagnosis including motor and sensory nerve conduction studies
•	Serum and urine monoclonal protein detection by immunofixation
•	Fasting blood glucose/Glycosylated hemoglobin (HbA1c)
•	Complete blood count
•	Renal function
•	Liver function
**If support for typical CIDP is needed**	Imaging of the brachial plexus and cervical nerve roots (US and/or MRI)
•	Cerebrospinal fluid examination including cells and protein
•	Nerve biopsy
**Proximal and distal sensory and/or motor deficit**	*Borrelia burgdorferi* serology
	HIV serology
	Serum vascular endothelial growth factor, ophthalmological and dermatological exam for POEMS
	HIV sorology
	Skeletal survey and calcium levels for multiple myeloma
	Nodal-paranodal protein antibodies
	Chr 17 dosage for *PMP22*
	RFC 1 test for CANVAS
	WES for other genetic neuropathies
	C-reactive protein
	Antinuclear antibody antibodies
**Multifocal/focal sensory and/or motor deficit**	Anti-PGL1, dermatological exam, mycobacterium PCR and bacilloscopy for leprosy
	Antinuclear antibodies, antineutrophil cytoplasmic antibodies, Erythrocyte sedimentation rate, C-reactive protein, nerve biopsy for vasculitis
	Anti-MAG antibodies when IgM monoclonal gammopathy is present
**Motor deficit**	Repetitive stimulation in low and/or high frequency and anti-VGCC or anti-ACHR or anti-Musk if neuromuscular junction abnormalities
	Anti-GM1 IgM antibodies if multifocal motor neuropathy is suspected
	Creatine kinase, lactate and aldolase levels, myositis specific autoantibodies, muscle MRI, and muscle biopsy if inflammatory myopathy is suspected
	WES for genetic neuropathies, myopathies, and neuromuscular junction disorders
**Sensory Deficit**	Antiganglioside antibodies
	Vitamin B12, B6, homocysteine levels
	Paraneoplastic antibody screen
	Somatosensory evoked potentials when nerve conduction studies are normal
	Sjogren assessment: anti-Ro, anti-La, Schirmer test, salivary gland biopsy
	Anti-FGFR3
	WES for sensory neuro/neuronopathies

Abbreviations: CANVAS, cerebellar ataxia with neuropathy and vestibular areflexia syndrome; CIDP, chronic inflammatory demyelinating polyradiculoneuropathy; IgM, immunoglobulin M; MAG, myelin-associated glycoprotein; MRI, magnetic resonance imaging; PCR, polymerase chain reaction; PGL-1, phenolic glycolipid 1; *PMP22* , *peripheral myelin protein 22* gene; POEMS, polyneuropathy, organomegaly, endocrinopathy, monoclonal protein, and skin changes; RFC 1, 1, replication factor complex 1; US, ultrasound; WES, whole exome sequencing; Chr 17, chromossome 17; Musk, muscle specific kinase; anti-GM1, ganglioside IgM antibodies; FGFR3, fibroblast growth factor receptor 3.

Additionally, objective treatment response and nerve biopsy may also be used as supportive criteria to upgrade a diagnosis of “possible CIDP” to “CIDP” when clinical and electrodiagnostic criteria alone are insufficient. 2 These significantly reduces the rate of false positives, particularly in early diagnosis; they demonstrate a sensitivity of 83.3% for “CIDP” and 93.3% for “CIDP” or “possible CIDP,” along with a specificity of 94% for “CIDP” and 79% for “CIDP” or “possible CIDP” respectively. 32

### Genetic mimics of CIDP?

Clinicians should be aware that some hereditary neuropathies can present overlapping clinical or electrodiagnostic features with CIDP or its variants. A clear family history is a strong indicator of this possibility. However, up to 12.3% of the cases of apparently-sporadic axonal neuropathy present have disease-causing variants in genes associated with Charcot-Marie-Tooth disease (CMT). 33 In suspected demyelinating forms of CMT without positive family history, the frequency of duplication of the *peripheral myelin protein 22* ( *PMP22* ) gene ranges from 20 to 90% of the cases. 34 35

In patients with a slowly-progressive course, no proximal weakness, and uniform reduction in conduction velocities (CVs), a genetic cause should be always considered, even in the absence of affected family members. It is important to note that some forms of CMT may present with non-uniform CV and TD, 36 37 38 39 making differentiation even more challenging. The possibility of concomitance of CIDP and an inherited neuropathy has already been described in text. Additionally, unresponsiveness to appropriate immunotherapy is a common red flag observed in patients with hereditary neuropathy who are misdiagnosed as having CIDP.

In this context, hATTR should be considered, as it can closely mimic CIDP, particularly in late-onset sporadic cases occurring after the age of 50 years. 40 Symptoms such as prominent pain, dysautonomia, early-onset distal upper limb motor deficits, extension of small fiber sensory loss above the wrist, and absence of ataxia are uncommon in CIDP. A study 41 evaluated 150 patients with confirmed hATTR, and 32% of them had been misdiagnosed, with 61% of those cases initially thought to have CIDP.

In Brazil, a study 42 conducted a genetic evaluation of the *transthyretin* ( *TTR* ) gene in 119 patients diagnosed with CIDP and identified pathogenic variants in 5 individuals: 3 with the pVal30Met mutation, 1 with the p.Val71Ala mutation, and 1 with the p.Asp38Tyr mutation.

Additionally, the same cohort was analyzed 43 regarding variants in the *PMP22* , *gap junction protein beta 1* ( *GJB1* ), *myelin protein zero* ( *MPZ* ), and *lipopolysaccharide-induced TNF factor* ( *LITAF* ) genes: pathogenic variants were identified in 7 patients (5.9%), including 2 cases of *PMP22* duplication, 2 single nucleotide variants (SNVs) in *MPZ* , and 3 SNVs in *GJB1* . Combined, these findings indicate that 8.4% of the patients previously diagnosed with CIDP in this cohort presented a hereditary neuropathy. 43

### Which acquired conditions should be considered in the differential diagnosis?

Various acquired conditions can present with similar clinical features as CIDP ( Table 7 ). The presence of a very slowly-progressive neuropathy, intense pain, early muscle wasting, sphincter disturbance, cranial nerve involvement, head drop, respiratory disturbance, and/or systemic and autonomic symptoms are important reg flags for CIDP that should suggest the consideration of an alternative diagnosis. 44 In most cases, the laboratory evaluation should include complete blood count, assessment of electrolyte (including fasting glucose) levels, liver function tests, serum and urine monoclonal protein studies including immunofixation, thyroid function tests, assessment of angiotensin converting enzyme levels, testing for the human immunodeficiency virus (HIV) and human T-lymphotropic virus types I and I (HTLV-I/II), test for emerging viruses (including arboviruses and severe acute respiratory syndrome coronavirus 2, SARS-CoV2), hepatitis panel, and rheumatological laboratory test results.

**Table 7:** Differential diagnosis of CIDP according to clinical approach

Symptom distribution	Modalities impaired	Condition	Diagnostic clues and pitfalls
**Proximal and distal**	**Sensory and/or motor**	HIV	Monophasic progressive and good response to corticosteroids 44
		CISMP	Proximal predominant, normal conduction with abnormal sensory evoked potential and/or prolonged F-wave 45
		NF-186/140	Acute/subacute, usually severe respiratory failure 46
		PanNF	Acute, aggressive, rapidly-progressive, refractory with autonomic and respiratory failure 46
		CNTN1	Acute/subacute, late onset, associated nephropathy, tremor, sensory ataxia, and distal weakness 46
		CASPR	Acute/subacute, neuropathic pain 46
		NF-155	Early onset, tremor, high CSF protein, early axonal loss, good response to rituximab 46
		Multiple myeloma	Bone osteoblastic lesion, hypocalcemia, renal impairment 47
	**Motor > sensory**	Porphyria	Abdominal pain, psychiatric disturbance, hyponatremia, seizures 48
		CIMP	Almost normal conduction study, abnormally-prolonged F-wave 45
	**Sensory > motor**	CISP	Sensory ataxia and normal sensory conduction with abnormal sensory evoked potential 45
		CANOMAD	Ophthalmoplegia, IgM gammopathy with cold agglutinin and disialosyl antibodies 47
		CANDA	Equal CANOMAD without ophtalmoplegia 47
		RFC1	Late onset, eventually with cerebellar ataxia and/or vestibular impairment 49
		Syphilis	Late manifestation, possibly negative VDRL and positive FTAbs 50
		Paraneoplastic	Rapidly-progressive cognitive impairment, cerebellar ataxia, and macular degeneration 51
		Sjogren	Sicca syndrome, small fibre neuropathy 51
		Vitamin B12 deficiency	Megaloblastic anemia, increased homocysteine levels 52
		Toxic	Check Vitamin B6 use and cisplatin 52
		Copper deficiency	Zinc supplementation/intoxication 52
Distal > proximal	**Sensory and** **motor**	POEMS	Early axonal loss, skin lesions, increased VEGF, osteoclastic lesion 47
		hATTRv	Positive family history, cardiopathy, carpal tunnel syndrome, mainly axonal with only a hint of demyelination 53
		AL-Amyloidosis	Macroglossia, periorbital ecchymosis, autonomic failure, nephrotic syndrome 47
		Other genetic	PMP22 dup, SIGMAR1, FIG4, MPZ, CMTX, Refsum, Krabbe, NEFL, PLEKGH5, SH3TC2 39 54
	**Sensory > motor**	Anti-MAG	Terminal latency index < 0.26 47
Multifocal/Focal	Sensory and motor	Vasculitis	Pain in nerve trajectory, increased inflammatory markers 52
		Diabetic radiculoplexopathy	Asymmetrical painful atrophy predominating in the lower limbs 55 56
		Poorly controlled diabetes	Distal motor conduction velocity slowing; high HB1AC; may cause albumin-cytologic dissociation 55
		Entrapment	Common sites: carpal tunnel, cubital tunnel, head of the fibula 55
		HNPP	Positive family history and eventually prolonged distal motor latencies on nerve conduction study 54
		Benign peripheral nerve tumor	Slowly progressive or static with abnormal nerve imaging enhancement 57
		Lymphoma	Painful, rapidly-progressive, abnormal nerve imaging enhancement 47
		Neuralgic amyotrophy	Pain preceding symptoms, non-myotome atrophy and upper-limb predominant 58
	**Motor > sensory**	Multifocal motor neuropathy	Upper-limb predominant, nerve trajectory, conduction block, possibly positive anti-GM1 54
	**Sensory > motor**	Leprosy	Thermo-dependent, fascicular, patchy demyelination in non-common entrapment sites 59 60

Abbreviations: CANDA, chronic ataxic neuropathy and disialosyl antibodies; CANOMAD, chronic ataxic neuropathy, ophthalmoplegia, immunoglobulin M paraprotein, cold agglutinins, and disialosyl antibodies; CASPR, contactin-associated protein; CIDP, chronic inflammatory demyelinating polyradiculoneuropathy; CIMP, chronic inflammatory motor polyradiculopathy; CISMP, chronic inflammatory sensorimotor polyradiculopathy; CISP, chronic inflammatory sensory polyradiculopathy; CNTN1, contactin 1; CSF, cerebrospinal fluid; hATTRv, hereditary transthyretin-related amyloidosis; HB1AC, glycated hemoglobin; HNPP, hereditary neuropathy with liability to pressure palsies; MAG, myelin-associated glycoprotein; NF, neurofascin; *PMP22* , *peripheral myelin protein 22* gene; POEMS, polyneuropathy, organomegaly, endocrinopathy, monoclonal protein, and skin changes; RFC 1, replication factor complex 1; VEGF, vascular endothelial growth factor; FTA-Abs, fluorescent treponemal antibody absorption test; AL amyloidosis, amyloid light chain amyloidosis; PMP22 dup, PMP22 duplication.

Diabetic neuropathy is highly prevalent, and there is evidence suggesting it may increase the risk of developing CIDP or even that diabetic neuropathy may be misdiagnosed as CIDP. 15 61 62 Nerve conduction studies occasionally show demyelinating features that fulfill the electrophysiological criteria for CIDP. This is particularly common in diabetic patients with persistently-high glycemic levels, which can lead to transient slowing of CV. 63 In known diabetic patients, capillary blood glucose should be measured prior to the NCS. If glucose levels are significantly elevated and CVs are reduced, the examination should be repeated under conditions of normalized glycemic levels. These patients usually complain of intense neuropathic pain and have no proximal weakness on neurological examination.

Some immune-mediated conditions, such as systemic lupus erythematosus (SLE) and Sjögren's syndrome, can rarely present as CIDP-like neuropathy. Additionally, sarcoidosis-associated CIDP presents with atypical features such as older age at onset, weight loss, unresponsiveness to intravenous immunoglobulin (IVIg), and the need for specific management strategies. 64

Some infectious disorders, such as chronic HIV infection, may cause an immune-mediated demyelinating process in the PNS, but, different from classic CIDP, the patients usually present a slowly-progressive course, and the CSF has lymphocytic pleocytosis. Another common infectious neuropathy that may mimic CIDP is primary neuritic leprosy (PNL). In this rare type of leprosy, the patients do not present skin lesions, and the NCSs usually present focal slowing of the CV with or without TD. These patients do not present proximal weakness but are misdiagnosed as having focal/multifocal CIDP. 60

Another group of disorders are the paraproteinemic neuropathies, which may or may not be related to underlying hematological malignancy, and are associated with a relative risk of 5.9% (95%CI: 1.2–28.4) of developing CIDP. 65 All patients with slowed CV and suspected acquired neuropathy need to be investigated for paraproteinemic neuropathy. 47 Both immunoglobulin M (IgM) and non-IgM paraproteins can mimic CIDP. In the IgM group, distal acquired demyelinating sensory (DADS) is the most common in the clinical practice, and it is related to the presence of anti-myelin-associated glycoprotein (anti-MAG) antibody. Distal motor latencies are usually very prolonged, distinguishing it from CIDP. In the absence of detectable IgM M-protein, it should be managed as distal CIDP.

In the non-IgM group, polyneuropathy, organomegaly, endocrinopathy, monoclonal protein, and skin changes (POEMS) syndrome is frequently misdiagnosed as CIDP, especially at early stages, in which the classic features may be absent. Diverging from CIDP, it usually presentes with severe pain and early axonal degeneration that early impairs the ability to walk. Conduction block and TD are uncommon on routine NCSs. The inclusion of the serum level of vascular endothelial growth factor (VEGF) as a major criteria for POEMS syndrome significantly increased the specificity for its diagnosis. To exclude POEMS syndrome, a skeletal bone survey (to look for osteosclerotic myeloma) or positron-emission tomography-computed tomography (PET-CT, to look for osteosclerotic myeloma or Castleman disease) along with the assessment of VEGF levels are required. 47 66 67

Finally, acute-onset CIDP is usually misdiagnosed as GBS, and it should be considered when the symptoms progress for longer than 8 weeks, and when there are more than 2 clinical relapses. All of the aforementioned conditions can present with overlapping symptoms, and distinguishing them from CIDP is critical for the appropriate treatment. The presence of specific clinical features, serological markers, and electrophysiological findings can aid in differentiating these conditions from CIDP ( Table 7 ).

### How and when to start treatment?

The goal of CIDP treatment is to improve function by addressing weakness and sensory loss, while achieving and maintaining long-term remission without overtreating the patient. It is strongly recommended that CIDP diagnosis and the treatment regimen be managed preferably by experts in inflammatory neuropathies due to the overall disease burden, the high number of false positives, the substantial costs of treatment, the potential for significant iatrogenic side effects of immunosuppression, and the need for specialized knowledge of neurophysiological aspects, clinical course, and disease variants.

The immunotherapy for CIDP should be started whenever a patient develops significant motor involvement and functional disabilities. Patients with mild sensory symptoms and no functional deterioration could be followed up clinically. The treatment has two stages: induction and maintenance.

### What are the first-line treatments?

The first-line treatments for CIDP include corticosteroids, IVIg, and plasmapheresis (or plasma exchange, PLEX). There is no consensus nor unequivocal level-I evidence to support one of these options. Intravenous immunoglobulin is the only one with high-quality placebo control evidence to support short-term and sustained improvement. Previous studies with corticosteroids had different designs, which hinders a comparison of their outcomes. However, there is strong clinical evidence 68 69 supporting similar efficacy of corticosteroids and IVIg for typical CIDP management. Despite that, the EFNS/PNS consensus 2 and a Cochrane meta-analysis 69 suggest IVIg may present the best therapeutic index.

The clinical decision should be based on cost, local resources, availability, disease phenotype, and overall clinical status (such as presence of diabetes, renal function, IgA deficiency, age or evidence of risk of side effects from a given agent). Human immunoglobulin is not included in the list of drugs offered by SUS for the treatment of CIDP in Brazil. Given the limited availability and the high costs, it is reasonable to recommend steroids as the first-option treatment for typical CIDP in treatment-naïve patients, provided there are no contraindications, such as uncontrolled diabetes.

Corticosteroids have been used to treat CIDP since the late 1950s, with an efficacy rate ranging from 30 to 90%. 70 They act by suppressing multiple genes involved in the inflammatory process while also activating the transcription of anti-inflammatory genes. Corticosteroids are recommended as the first option in almost all international guidelines. 68

There is limited information regarding the optimal steroid regimen. The options include daily oral prednisolone (60 mg/day), a high monthly dose of oral dexamethasone (40 mg/day for 4 consecutive days) or monthly IV methylprednisolone (in doses of 0.5g/day for 4 days to 1g/day for 3 to 5 days). 68 70 71 A low-quality study 72 showed no difference in effectiveness among the three regimens. A moderate-quality study showed no difference in efficacy between high monthly doses of oral dexamethasone compared with daily doses of oral prednisolone for 6 months. Both monthly IV or oral steroid regimens are associated with fewer side effects compared to daily oral steroids. 68 73 74 Some studies 73 74 suggest that pulsed steroids may lead to a higher probability of disease remission at 6 months than IVIg or oral steroids.

Different forms of CIDP may respond variably to treatment, with some more likely to improve with steroids, while others show better outcomes with IVIg. Focal and multifocal variants are more likely to improve with IVIg treatment compared to steroids. The efficacy of the steroid regimen should be evaluated after 3 months. If disease remission is not achieved, a switch to IVIg should be considered. Up to 1 in every 4 patients with CIDP under steroid therapy achieves either remission (defined as being asymptomatic for < 5 years without treatment) or cure (defined as being asymptomatic for > 5 years without treatment). 71

In centers where IVIg is available, it can be used as a first-line therapy or as a rescue therapy for those who have failed an adequate steroid regimen or have known contraindication to steroids. Intravenous immunoglobulin acts on the complement system; 75 the neonatal fragment crystallizable (Fc) receptor (FcRn) recirculating immunoglobulin G (IgG) and increasing its half-life possibly competes for the binding site in nerves with autoantibodies that are probably present in patients with CIDP. 76

The induction stage of the IVIg treatment involves an initial loading dose of 2 g/kg, administered over 2 to 5 days, with a daily maximum dose of 80 g/day to reduce the risk of adverse events. 68 70 71 The patients should be clinically evaluated 6 weeks after: if there is partial or no improvement, a second loading dose of 2 g/kg, administered in the same manner, should be provided, and the patient, assessed after another 6 weeks. For patients with a positive response, the maintenance stage is recommended, which consists of the administration of 1 g/kg of IVIg every 3 weeks. About 15% of the CIDP patients need only 1 to 2 courses of IVIg to reach remission. Subcutaneous immunoglobulin is an option for maintenance therapy, but it has not been adequately studied for the induction stage. However, if the patient shows no clinical response after the second loading dose, maintenance therapy is not recommended. Comparative studies with corticosteroids did not show a significant difference in efficacy, although IVIg was slightly superior. However, two studies 68 71 showed that treatment withdrawal was more successful after the use of corticosteroids.

5 randomized clinical trials (RCTs) totalizing 235 subjects (mainly treatment-naïve) were carried out comparing IVIg with placebo, 1 RCT compaired it with PLEX and 3 compaired it with corticosteroids. Intravenous immunoglobulin was effective and well tolerated in the short and long terms. 70 It was also more effective than placebo in reducing disability, 68 70 71 but with more mild adverse events and no difference regarding serious side effects compared to controls. 68 A Japanese study 77 (n = 49) also reported long-term efficacy (induction IVIg dose of 2 g/kg for 5 consecutive days, maintenance IVIg dose of 1.0 g/kg every 3 weeks for up to 52 weeks). The study 77 reported a response rate of 78% at 28 weeks, with a 10.5% relapse rate within the population that continued treatment until week 52. The predictors of good response include absence of pain, rapid progression after onset, a relapsing–remitting and monophasic disease course, female gender, similar weakness in the upper and lower limbs, more than twofold increase in CSF protein levels, presence of CB on EMG, and lack of muscle atrophy. 78 In the IVIg for CIDP (ICE) trial, ^88^ most IVIg-dependent patients deteriorated within 4 months of stopping treatment, which reflects an active disease. 79 A study 80 showed that the switch from IVIg to subcutaneous immunoglobulins (SCIgs) in CIDP and multifocal motor neuropathy (MMN) patients, thus achieving more constant serum levels due to weekly SCIg infusions, was not as efficacious as IVIg in a subset of patients. Case studies, small, short-term, randomized, and controlled trials, as well as open-labelled long-term observations all indicate that maintenance treatment with SCIg can preserve muscle strength and function in patients with CIDP and MMN. The optimal SCIg dosage might be slightly higher than the one used for the IVIg therapy. In general, the side effects seem to be restricted to local reactions at the infusion site. Quality of life scores seem to improve when compared with intravenous infusion during hospitalization, and most patients switched to SCIg therapy prefer to continue their original regimen. 81 82

Most centers do not consider PLEX an initial alternative due to the need for administration in specialized facilities and its longer duration. Instead, it is often reserved for cases with rapid deterioration, 71 or in cases that do not respond to corticosteroids and IVIg. 68 It removes antibodies and other small molecules, such as cytokines, that affect the function of T lymphocytes. The protocol for acute exacerbations or refractory patients includes 5 sessions on consecutive or alternate days during the induction stage, whereas the maintenance therapy includes just 1 or 2 sessions, interspersed at intervals of a few weeks (2–4). Two RCTs were performed with sham exchange, showing superiority of PLEX. 68 A moderate-quality RCT showed no difference in efficacy between PLEX and IVIg. 68 There is no RCT comparing PLEX with corticosteroid therapy.

### What are the advantages and disadvantages of the main proposed treatments?

Both IVIg and PLEX are quite expensive and require greater care for their implementation; corticosteroid therapy, on the other hand, is much cheaper and accessible, but it results in more adverse effects. 70 71 In most countries, PLEX is cheaper than IVIg, but it is not available in most Brazilian cities. The common complications of PLEX include hypocalcemia, hypotension, bleeding, infections, and trauma resulting from the passage of the central venous access when there is no peripheral access available.

As for the IVIg side effects, they may be related to the infusion, especially headache, nausea, skin rash, flu-like symptoms, and even signs of meningeal irritation. Serious and long-lasting adverse events, on the other hand, are rare (0.04%): renal tubular necrosis, thromboembolic events, aseptic meningitis, hemolysis, and anaphylaxis. A predictor of adverse events is renal failure, and attention is recommended when administering IVIg to patients with a high level of creatinine. 83

The adverse events related to the prolonged use of corticosteroids include depression, weight gain, osteoporosis and hyperglycemia, gastric ulcers, necrosis of the femoral head, and others. 84

**Figure 1:**
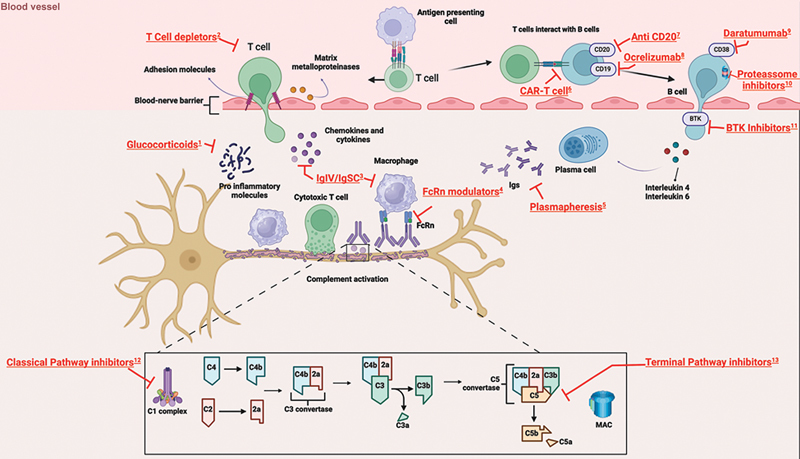
Overview of treatment targets in chronic inflammatory demyelinating polyradiculoneuropathy (CIDP). ( **1** ) Glucocorticoids act through non-genomic and genomic effects, leading to increased production of anti-inflammatory proteins and reduced proinflammatory proteins; ( **2** ) T-cell depletors: azatyoprine, cyclophosphamide, mycophenolate mofetil, autologous hematopoietic stem cell transplantation (ASCT), tacrolimus, cyclosporine, and methotrexate act through T and B cell depletion/modulation; ( **3** ) immunoglobulin acts through multiple ways: modulating B cells, reducing antibody production, neutralizing pathogenic antibodies, inhibiting complement, suppressing macrophage-mediated injury, downregulating the production of inflammatory cytokines, and inhibiting antigen-presenting cells, as well as cellular cytotoxicity; ( **4** ) neonatal fragment crystallizable receptor (FcRn) modulators: efgartigimod, batoclimab, nipocalimab, and rosanolixizumab reduce the binding of pathogenic antibodies to the FcRn, thus reducing the protective effect of the FcRn against lysosomal degradation, leading to autoantibody depletion; ( **5** ) Plasmapheresis primarily removes pathogenic autoantibodies from the blood circulation; drugs acting through B-cell depletion/modulation ( **6–11** ): ( **6** ) chimeric antigen receptor (CAR) T cell; ( **7** ) anti-cluster of differentiation 20 (anti-CD-20): rituximab, oftamumab, and ublituximab; anti CD-19: ( **8** ) anti-CD-19 ocrelizumab; ( **9** ) anti-CD-38 daratumumab; ( **10** ) proteassome inhibitors: bortezumib, carfilzomib, and ixazomib; (11) Bruton's tyrosine kinase (BTK) inhibitors: ibrutinib and zanubrutinib; ( **12** ) classic pathway inhibitors: SAR445088 and GL-2045; and ( **13** ) terminal pathway inhibitors: eculizumab, ravulizumab, and zilucoplan: act through complement inhibitiom, selectively blocking downstream complement activation involved in the inflammatory processes, causing demyelinating damage.

An overview of treatment targets in CIDP is shown in Figure 1 .

### What are the second-line therapies available?

Various immunosuppressive and immunomodulatory drugs, including azathioprine, cyclosporine, cyclophosphamide, methotrexate, and mycophenolate mofetil, have been used as second-line treatments for CIDP in patients who are refractory to or intolerant of the first-line therapies. However, their efficacy in CIDP is often based on anecdotal evidence or small, non-randomized studies. Patients requiring long-term treatment to maintain remission may benefit from the second-line therapies, as they can reduce the need for IVIg or corticosteroids. The use of monoclonal antibody targeting cluster of differentiation 20 (CD20) on B cells (rituximab) has shown promising results, 84 especially in patients with CIDP associated with other autoimmune conditions or those with a relapsing-remitting course; however, large randomized controlled trials are needed to establish its efficacy and safety in CIDP. Other agents, such as interferons and etanercept, have been explored, 84 but their efficacy remains unclear, and they are not widely used for CIDP in the clinical practice. Subcutaneous efgartigimod, an FcRn inhibitor, was recently evaluated in the ADHERE study, 85 a multistage, double-blinded, placebo-controlled trial, enrolling participants with CIDP from 146 clinical sites from Asia–Pacific, Europe, and North America. The results were considered promising, but we are waiting for a longer period of observation in controlling CIDP symptoms and its real cost-effectiveness. Likewise, regarding, Batoclimab, another FcRn inhibitor used for CIDP treatment, we are also waiting for reports of its long-lasting efficacy and safety. 84 86 The use of these therapies is often guided by the need to balance potential benefits with the risk of adverse effects, which can be significant with immunosuppressive drugs.

### What are the clinical scales to be applied to monitor CIDP treatment?

Subjective perception of disease progression or treatment benefits reported by patients should be considered with caution. Clinical tools have been proposed for the assessment of CIDP patients, considering clinical features and the effects of the treatment on daily life activities. Impairment in QoL, sensory deficits, weakness, and overall disability are the primary outcomes measures evaluated by these instruments.

Considering that none of the instruments has demonstrated superiority over the others to detect significant clinical changes, 87 we recommend a combination of instruments whose application is feasible in the clinical practice and that have also been validated in a CIDP population, including the the INCAT criteria, 88 the INCAT Sensory Subscore (ISS), the Overall Disability Sum score (ODSS), the Rasch-Built Overall Disability Scale (R-ODS), and the Medical Research Council sum score (MRC), which we will detail briefly, as follows: 89

- INCAT: a 10-point ordinal measure capturing problems in daily arm and leg activities and mobility, it was chosen as the primary outcome in the largest trial performed in patients with CIDP, the ICE study; 88
- ISS: devoted to the evaluation of sensory deficits, it encompasses two-point discrimination, pinprick, and vibration sense. Its scores vary from 0 (normal evaluation) to 20 (maximal sensory impairment); 89
- ODSS: this instrument was developed to assess treatment efficacy for immune-mediated neuropathies. It is based on the 10-point INCAT disability score; therefore, it evaluates the upper and lower limbs, whose scores are based on the patients' level of disability and range from 0 to 12 points (0–5 for the upper limbs and 0–7 for the lower limbs), with a score of 12 indicating the worse scenario; 89
- R-ODS: as a patient-based tool that considers the limitations in activity and social participation, this 24-item survey offers to each of them a 3-option response with a range of 0 to 48 with a nomogram translation to 0 (most severe limitations in activity and social participation) to 100 (no limitations in activity and social participation); 89
- MRC: one of the most widely-known strength scales, the MRC grades from 0 to 5 the strength of 6 muscle groups bilaterally: arm abductors, forearm flexors, and wrist extensors in the upper limbs, and leg flexors, knee extensors, and dorsal foot flexors in the lower limbs (range: 0–30). 89

Considering the aforementioned scales, it would take 10 to 15 minutes on average to evaluate a CIDP patient. Several other different clinical instruments may be useful and feasible on clinical grounds in the daily practice. We opted not to include QoL or pain-focused scales, but this should not hinder neurologists from tailoring their practice and using them to make their evaluation more comprehensive. However, it is not an exaggeration to stress that, no matter how comprehensive the set of clinical scales is, it should not detain a careful neurological with a meticulous record of findings.

### In what situations should we be suspicious when the patient does not respond to treatment?

Patients with CIDP who do not respond to treatment should always be reevaluated considering misdiagnosis; inadequate immunotherapy; atypical and seropositive forms; late disease stage with irreversible secondary axonal loss; and refractoriness (active but non-responding disease).

### Misdiagnosis

The lack of responsiveness to the first-line treatment and/or rapid deterioration of the disease should prompt a reconsideration of the CIDP diagnosis. 90 There are several possible explanations for the high rate of CIDP misdiagnosis. Like many diseases without a diagnostic biomarker, the diagnosis of CIDP requires the combination of multiple clinical and laboratory components. The clinical spectrum of CIDP is very heterogeneous (only 50% of the patients present with a classic form), and the commonly-applied criteria for CIDP are only about 80% specific. Moreover, “CIDP Variants” includes patients with a large variety of clinical manifestations, with pure motor, pure sensory, asymmetric, focal, and distal predominant clinical features, often providing diagnostic liberties to diagnose unconfirmed CIDP, especially in patients with poorly-defined chronic symptoms such as pain and fatigue. The main clinical pitfalls related to misdiagnosis are absence of ankle reflexes alone, no reduction in the vibration sense, and response to immunotherapy that is subjective or not specific. Moreover, electrodiagnostic errors have been found to be a major factor in misdiagnosis. 9 A common finding among misdiagnosed patients is that they tend to present only mild or moderate signs of demyelination, which were nestled within a principally axonal damage. 9 Furthermore, homogeneous nerve conduction slowing and abnormalities exclusively in lower limbs nerves should raise suspicion regarding the diagnosis.

### What about inadequate immunotherapy?

In general, 50 to 70% of naïve CIDP patients respond to one of the first line-immunotherapy treatments. An additional 50% of non-responding patients improve when a second treatment is implemented, resulting in the prevalence of an overall 80% of responsiveness rate with at least 2 immunotherapies. 91 Even more, 20 to 33% of CIDP patients remain refractory to IVIg, PLEX, and corticosteroids. A study 90 reported that 87% of the patients with confirmed refractory CIDP showed improvement with the escalation of therapy, suggesting that inadequate immunosuppression may be one of the reasons for therapeutic failure. Although CIDP is amenable to treatment with immunotherapy, it is difficult to predict the likelihood of a response in an individual patient. Various factors have been suggested as predictors of outcome and/or treatment response, including the rate of disease onset, the pattern of weakness, the presence/absence of pain, the disease course (such as progressive, relapsing), the duration of the disease, the presence/absence of monoclonal gammopathy, the pattern or degree of demyelination, the extent of axonal loss, and electrophysiological criteria. 25 92

### How can the dose of immunoglobulin be adjusted?

Six weeks after the induction stage (two cycles), if there is no clinical response, IVIg maintenance therapy should not be initiated. However, if there is a positive response, characterized by a tendency towards remission, or if there has been any objective improvement (based on clinical scales), maintenance should be started. 93

The ICE study, 88 94 which involved 117 patients, showed that, after the initial IVIg dose of 2g\kg in 2 to 4 days, followed by 1g\kg in 1 to 2 days at intervals of 3 weeks for 6 months to 1 year, the patients presented objective improvement, with decreased disability and decreased relapses, when compared to those receiving placebo. A retrospective study 78 found that 76% (214 out of 281) of CIDP patients responded to treatment with immunoglobulin. A meta-analysis 6 of 4 randomized and controlled double-blinded studies with 235 participants enabled us to indicate the dose of 2g\kg that produces an improvement in disability in 2 to 6 weeks (class-1 evidence and level A of recommendation). The dose will always be individualized, and if the disease is stable, the dose or frequency will be reduced (20% of the total dose per session until 0.4g/kg per cycle), until it can be established if the patient still needs the infusions and how often. It is estimated that 30% of the patients can achieve remission without further need for IVIg. 95

### Variants and seropositive forms?

Other studies 96 have supported our position that some atypical forms and seropositive CIDP may be less respondent than classic CIDP. The different responses to the immune treatments may depend on the different pathophysiology of each CIDP subtype, and some factors could determine treatment response and outcomes. Among the whole CIDP spectrum, distal acquired demyelinating sensory neuropathy (DADS) and Lewis Sumner Syndrome seem to respond poorly to the first-line therapies and may require alternative options of treatment.

### Late disease stage with irreversible secondary axonal loss?

The only factor that differed significantly between patients who responded or not to treatment for CIDP is the duration of neuropathy, which is probably related to the degree of axonal degeneration. The identification of patients with active but non-responding disease (refractory CIDP) is crucial to differentiate them from those who have inactive disease with chronic stable deficits, probably with axonal loss and an irreversible clinical condition.

### Refractory CIDP?

Approximately 10% of these patients will not respond to any treatment despite an adequate trial of treatment and use of different first-line drugs. Currently, there are no specific biomarkers for treatment responsiveness in individual patients, and it can be difficult to predict which patients are most likely to respond. Refractory CIDP will require escalation of treatment with other immunosuppressant drugs. 2

### Cost and use of medical assistance?

Incorrect diagnosis results in inadequate and often prolonged corticosteroid and IVIg therapy, with a potential financial burden on the healthcare system. The use and costs of healthcare resources are substantial for CIDP patients. In 2011, a study analyzed insurance claims data for 73 CIDP patients among 6.5 million lives covered in 9 commercial health plans in the United States. The annual health plan cost per patient was of almost US$57,000. Pharmacy claims were the main cost driver, representing 57% of health plan costs. Only 49% of the patients received immunomodulatory treatment for CIDP, including IVIg (26%), prednisone (16%), and immunosuppressants (7%); 2 patients received PLEX. Intravenous immunoglobulin accounted for 90% of the drug costs, with an mean cost of US$108,016 ± US$18,437 per patient. 97

Different studies on autoimmune diseases have analyzed the cost-effectiveness of switching patients from regular IVIg administered in the hospital to home treatment with SCIg. These studies suggest that SCIg is more cost-effective, primarily due to the reduced need for professional supervision during the infusions. Furthermore, avoiding transportation to the hospital as well as loss of income during hospitalization further improves the cost-effectiveness of the SCIg therapy. In neurology, there is a lack of clear response regarding the cost-effectiveness of the SCIg regimen. Two small Italian studies 91 examined the impact of switching to SCIg in patients with CIDP and MMN. In one of these studies, the authors 91 performed a cost-minimization analysis comparing the direct costs of each treatment regimen.

### Reduction or withdrawal of medication?

Reducing or withdrawing treatment may pose a risk of relapse. Electrodiagnostic studies were performed on ICE study 88 participants who responded to treatment in the first period and were subsequently randomized again to placebo in the 24-week extension phase. These data indicated that an increase in the total number, specifically of CB, may signal an increased risk of relapse after treatment discontinuation, whereas the absence of new demyelination findings may suggest a decreased risk of subsequent relapse. 88

### What to do when the patient does not improve?

One of the biggest difficulties in caring for patients with CIDP is how to manage those who do not improve clinically with the treatment implemented, and at what point the change in therapy should be established when a patient does not improve after the first treatment regimen. A previously-mentioned multicenter retrospective study 78 of CIDP patients undergoing treatment showed that 214 (76%) out of 281 patients responded to IVIg. A total of 58 of the 67 nonresponders to IVIg were assigned to subsequent treatment with PLEX or corticosteroids: 16 (67%) out of 24 patients responded to PLEX, and 20 (59%) out of 34 responded to the corticosteroid treatment. Subsequently, a third method of treatment was initiated in non-responders in the PLEX and corticosteroid treatment groups: 6 (75%) out of the 8 PLEX non-responders responded to the corticosteroid treatment, and of the 4 corticosteroid non-responders submitted to PLEX, 3 (75%) responded. ^78^ Therefore, CIDP patients may still improve with the use of another proven effective treatment if the first-line treatment or subsequent treatment is ineffective. 93

Refractory CIDP is defined as a lack of clinical improvement after the use of standard treatments, considering the minimal duration of treatment and optimum dose. In most papers, there is no formal definition of what should be considered clinical improvement. Some authors 98 have used the modified Rankin scale (MRS) and considered improvement after treatment of at least one point to be clinically meaningful. Another strategy relies on the approach used in randomized clinical trials to define responders. 88 A patient is considered a responder when there is improvement of at least one point in the modified 10-point INCAT score. Hence, lack of improvement would be seen in patients presenting no change in any of those scales (MRC or INCAT) after treatment. Patients would be considered refractory if they failed at least 2 out of the 3 standard CIDP treatments (IVIg, steroids or PLEX). Regarding the duration of treatment, it has been proposed 98 that 3 months for each therapeutic agent would be the minimum period of evaluation. The dosages should be those routinely prescribed in the clinical practice. The patients' clinical conditions, adverse events, and presence of comorbidities are conditions that modify the therapeutic approach strategy for patients with CIDP.

### What are alternative treatments for patients who do not respond to first-line drugs?

The indications to consider alternative immunosuppressive agents for patients with CIDP are as follows:

- The patient has not improved with sequential or combined trials of corticosteroids, PLEX, and IVIg;
- The patient has improved with these treatments but has frequent relapses, usually with attempts at weaning the medication, making continued therapy cumbersome, time-consuming, and costly; or
- The patient has developed intolerable adverse effects with the proven therapies.

Immunosuppressive and immunomodulatory agents have been proposed for refractory CIDP non-responders to conventional therapies, including azathioprine, methotrexate, interferon beta-1a, cyclophosphamide, cyclosporine A, mycophenolate mofetil, fingolimod, etanercept, alemtuzumab, and autologous or allogeneic peripheral blood stem cell transplantation ( Figure 1 ). Most of the data available on such agents in CIDP derives from observational studies and randomized controlled trials, including azathioprine, interferon beta-1a, methotrexate and, recently, fingolimod, and have not shown that they are effective in CIDP non-responders. 99

Recent studies have identified CIDP variants that were refractory to IVIg and had selectively high immunoglobulin G4 (IgG4) class antibody titers against nodal and paranodal antigens (neurofascin-155 and contactin-1). Most of these patients had distal motor involvement, tremor and ataxia, with subacute and severe course of the disease. 100 A common feature in patients with IgG4 antibodies is poor response or no response to the IVIg treatment, which could be explained by an inability to activate complement pathways and bind to the Ig Fc receptor, 101 which comprises the IgG4 syndrome. These patients showed variable responses to rituximab, from no response (mainly in patients with long disease duration and severe axonal damage) to remarkably good responses, with clinical recovery and depletion of IgG4 antibodies. 102 The efficacy of rituximab in patients with antibodies against the paranodal proteins should be investigated in future clinical trials.

Randomized clinical trials have not shown that other immunosuppressive drugs are effective in the treatment of CIDP. However, in the routine neurological practice, there is a tendency to associate even more than one immunomodulatory agent with conventional therapy to reduce the need of long-term dependency on high-dose IVIg or corticosteroids. 103 Less potent immunomodulatory drugs (azathioprine, mycophenolate mofetil, and methotrexate) are frequently used for mild CIDP, and cyclophosphamide, for severe CIDP. 91 103 More trials of adequate size, dose, and duration–not only on older agents but on new ones, as they become available–are needed to determine whether immunomodulatory agents are beneficial in CIDP.

When a patient does not improve after the first treatment regimen, the diagnosis should be reconsidered because misdiagnosis is very common.

## SUMMARY

Key points of the recommendations:

- Diagnosis and differential diagnosis:
  CIDP is often misdiagnosed, with studies indicating high rates of overdiagnosis.
  Electrophysiological studies (NCS/EMG) are of paramount importance; nevertheless, different hereditary neuropathies and other acquired neuropathies may fulfill the CIDP neurophysiological criteria, highlighting the importance of a judicious clinical assessment.
  Imaging techniques (MRI/ultrasonography), CSF analysis, and nerve biopsy are not mandatory, but they might aid in the diagnostic process.
- First-Line treatment strategies:
  Corticosteroids, IVIg, and PLEX are the primary options.
  IVIg is the most effective approach, with stronger evidence, but cost and availability limit access to it in Brazil.
  Corticosteroids are recommended as the first choice in treatment-naïve patients in low-income regions.
• Treatment monitoring and response evaluation:
  The use of clinical scales (such as INCAT, ODSS, R-ODS, and MRC) to track disease progression and response is warranted.
  Up to 30% of the patients can achieve remission and discontinue the first-line treatment over time; hence the importance of continuous access to treatment withdrawal.
- Management of refractory cases:
  10 to 20% of CIDP patients remain refractory despite treatment, often due to misdiagnosis or irreversible axonal damage.
  Second-line therapies include diverse immunosuppressants options, such as B and T cell modulators.
  New biological agents like FcRn inhibitors and complement inhibitors have shown promising results but require further research and validation.
- Healthcare system and public health vonsiderations:
  CIDP treatment places a financial burden on Brazil's healthcare system (SUS).
  Standardized diagnostic and treatment guidelines are essential to improve equity and accessibility.

In conclusion, the ABN recommendations emphasize early and accurate diagnosis, personalized treatment selection, and cost-effective management tailored to Brazil's healthcare system. Emerging therapies hold promise for refractory CIDP cases, but further studies are needed.
